# Mapping arterial stiffness metabolic biomarkers: a bibliometric analysis

**DOI:** 10.3389/fmed.2025.1557731

**Published:** 2025-05-22

**Authors:** Bangwei Chen, Kent Frederick Wirawan, Li Luo, Jianguo Zhang, Tao Li

**Affiliations:** ^1^BGI Genomics, Shenzhen, China; ^2^School of Biology and Biological Engineering, South China University of Technology, Guangzhou, China; ^3^Department of Electrical Engineering, Faculty of Engineering, Universitas Indonesia, Depok, West Java, Indonesia; ^4^Department of Pathology, School of Forensic Medicine, Shanxi Medical University, Taiyuan, China; ^5^School of Public Health, Hebei Medical University, Shijiazhuang, China; ^6^BGI Research, Shenzhen, China; ^7^School of Basic Medicine, Hebei Medical University, Shijiazhuang, China

**Keywords:** arterial stiffness, metabolome, biomarkers, uric acid, fatty acids, bibliometric study

## Abstract

**Background:**

Metabolomics enables systematic quantification of small-molecule dynamics underlying cardiovascular pathophysiology, offering mechanistic insights into arterial stiffness. This study aimed to identify the scientific output related to metabolome in arterial stiffness.

**Methods:**

This study conducted a bibliometric analysis of publications (2000–March 2025) indexed in the Web of Science Core Collection using VOSviewer and Bibliometrix. Analyses spanned country/institution contributions, authorship networks, journal impact, and keyword/abstract trends.

**Results:**

A total of 1,654 original and review papers in English published in 550 different journals by 1,566 institutions were found. Over the past two decades, there has been a significant increase in the number of publications, with seminal work by Maksim et al. demonstrating metabolite associations with arterial stiffness, particularly oxidized low-density lipoprotein. The United States led with 246 articles (14.9%), followed by China (209, 12.6%) and Japan (134, 8.1%). Keyword analysis revealed saturation in advanced vascular aging research (elderly populations, hypertension, stroke), while early vascular aging studies—particularly in youth people—remained underrepresented. A frequency analysis of abstract words identified uric acid, eicosapentaenoic acid, and bile acids as potential metabolic biomarkers. Text-mining identified uric acid, fatty acids and bile acids as priority biomarkers, with unsaturated fatty acids (e.g., eicosapentaenoic acid, arachidonic acid) dominating mechanistic investigations.

**Conclusion:**

This first bibliometric profile of arterial stiffness metabolomics highlights fatty acid metabolism as a mature focus, contrasted by emerging opportunities in bile acid and gut microbiota-derived metabolite research. Bridging gaps in early vascular aging cohorts and understudied microbial-host metabolic pathways may unlock novel therapeutic strategies for vascular rejuvenation.

## 1 Introduction

Arterial stiffness is an independent pathogenic factor of cardiovascular disease (CVD), characterized by a decreased ability of an artery to expand (1). Pulse wave velocity (PWV) is the standard measurements of arterial stiffness and other methods such as augmentation index (AIx) and stiffness index have been developed (2). Studies have established associations between arterial stiffness and multiple clinical biochemical indicators (3, 4), including low-density lipoprotein cholesterol (LDL-C), glycated hemoglobin (HbA1c), and C-reactive protein (CRP), among others.

The emergence of metabolomic technologies has enabled researchers to investigate the critical roles of metabolites and metabolic pathways in arterial stiffness (5, 6). Research has shown that individuals with metabolic syndrome (MetS) tend to have higher arterial stiffness (7). Additionally, other metabolic regulators, such as insulin (8), nitric oxide synthase (NOS) (9), and growth hormone (10), have been implicated in increasing arterial stiffness. As research into the relationship between metabolomics and arterial stiffness has progressed, metabolites have been classified, and their pathways identified. Targeted and non-targeted metabolomic analyses have identified a series of metabolites from specific classes or pathways, including carnitine (11), triacylglycerol, and sphingomyelin (12), that are significantly associated with arterial stiffness. Exploring these metabolites offers valuable insights into the intricate relationship between metabolism and arterial stiffness.

Despite progress in understanding arterial stiffness—acknowledged in the *ESC Guidelines for the management of elevated blood pressure and hypertension*—its precise pathophysiology remains incompletely characterized (13). As a hemodynamic marker of subclinical cardiovascular disease, arterial stiffness enables risk stratification in asymptomatic populations lacking conventional diagnostic features (e.g., angina, ECG abnormalities). This highlights the critical need to dissect its role as a preclinical biomarker. Unraveling the complexity of arterial stiffness demands focused interrogation of pivotal metabolites. Deciphering bidirectional interactions between metabolic networks and vascular remodeling may reveal drivers of disease progression, facilitating the development of therapies targeting metabolic dysregulation to alleviate arterial rigidity. Consequently, a comprehensive metabolomics framework is essential to advance arterial stiffness research.

In this study, we aim to elucidate global research trends in metabolomics and arterial stiffness through bibliometric analysis of literature retrieved from the Web of Science Core Collection (WoSCC) database. We will systematically examine publication growth patterns (2000–March 2025), national contributions, active institutions, leading authors, preferred journals, most cited articles, keyword networks, and term frequencies in abstracts. This investigation seeks to map interdisciplinary connections between arterial stiffness and metabolites, ultimately aiming to identify specific metabolites requiring prioritized investigation to guide future mechanistic and translational studies.

## 2 Materials and methods

### 2.1 Data source and search strategy

The data were retrieved from the WoSCC on 03April 2025. The search strategy was divided into three different schemes. The first scheme (#1) follows the terms AB = (“vascular stiffness” OR “arterial stiffness” OR “aortic stiffness”), while the second scheme (#2) utilized the terms TS = (“metabolize” OR “metabolome” OR “metabolic”). The third scheme (#3) combined the results of the first two, using the formula #3 = (#1 AND #2), ensuring that at least one of the terms from both schemes appeared in the selected publications. Additional filters were applied to limit the results to articles and reviews published in English between January 2000 and March 2025. This process yielded a total of 1,654 records, which were subsequently subjected to bibliometric analysis. Data were provided in the Supplementary materials.

### 2.2 Data analysis

Bibliometric analysis was performed using Bibliometrix 4.3.3 (R 4.3.2) for quantifying publication trends, citation networks, and international collaboration patterns (14). VOSviewer (1.6.20) (15) generated keyword co-occurrence networks through its clustering algorithm, optimized for visual interpretation of large datasets. The complementary use of both tools leveraged Bibliometrix’s statistical rigor and VOSviewer’s network mapping capabilities, following established protocols (16, 17).

## 3 Results

### 3.1 Overview of publication status

**FIGURE 1:**
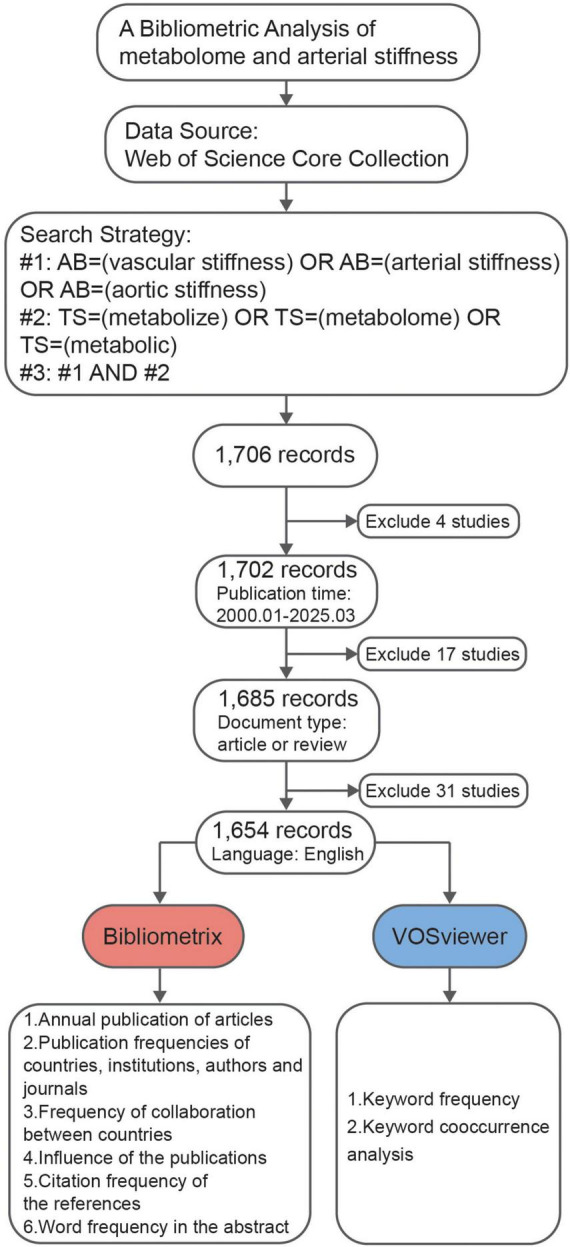
Workflow of the study.

**FIGURE 2:**
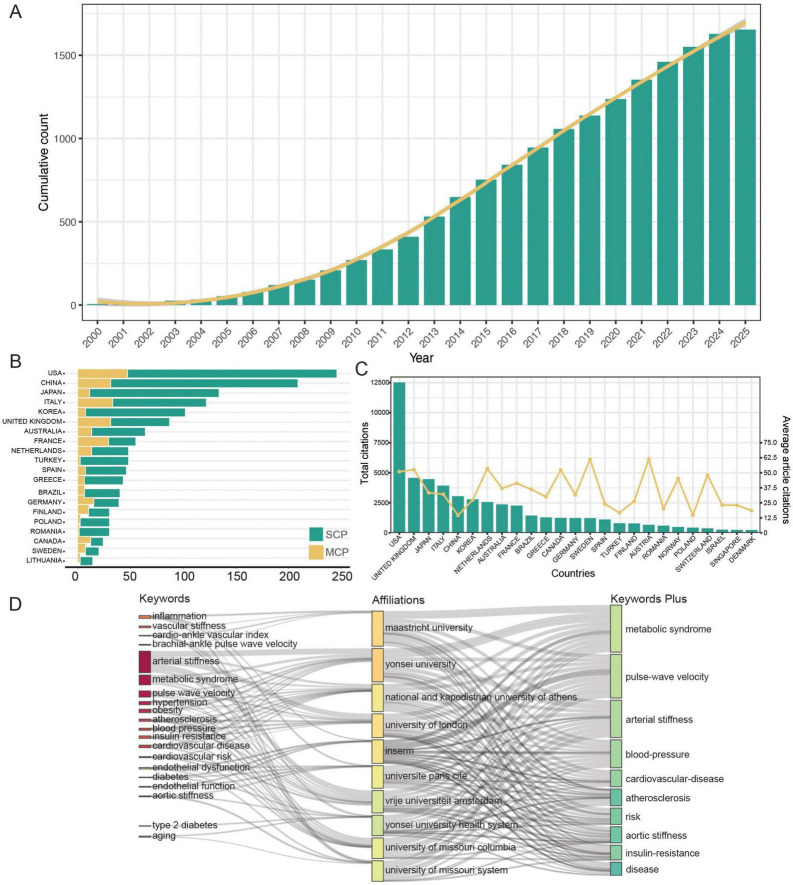
**(A)** The cumulative number of publications. **(B)** Publication counts based on corresponding author’s country. SCP, single country publications; MCP, multiple country publications. **(C)** The top 10 countries with the highest average citations. **(D)** Three-field plot of the keywords analysis on affiliations and keywords of publication (left field: keywords; middle field: affiliations; right field: keywords plus). INSERM, The National Institute of Health and Medical Research.

A total of 1,654 published articles were identified based on the search strategy (Figure 1). Despite some fluctuations, the number of related publications grew rapidly until 2013, after which it remained steady. After 2013, the cumulative publication curve seems to be linear (Figure 2A). These findings suggest that the field of metabolomics research in arterial stiffness is well-respected and has produced a substantial amount of literature.

According to corresponding authors, publications were contributed by 58 different countries, with 30 of these having 10 or more papers. The United States of America (USA) had the most publications (246, 14.9%), followed by China (209, 12.6%), and Japan (134, 8.1%). The USA leads the world in the number of international collaborative studies (*n* = 47, Figure 2B). The countries with the most prevalent international collaboration are Austria (63.3%), Iran (54.5%), and France (52.7%). In terms of citations, the USA has the most total citations (*n* = 12,514), while Sweden has the highest average citations (*n* = 61.3, Figure 2C).

A total of 1,566 institutions were associated with the literature, with 184 institutions publishing 10 or more articles. Yonsei University has the most research publications in this field, with 97 (6.2%), followed by Maastricht University with 90 (5.7%), and the French National Institute of Health and Medical Research (INSERM) with 85 (5.4%). Furthermore, the proportion of core topics for each institution was presented (Figure 2D), illustrating the associations and distribution among institutions and keywords. In general, the top 10 institutions contributed to the 20 topics represented by the keywords. The research on metabolomics and arterial stiffness at Yonsei University primarily focuses on arterial stiffness-related diseases such as hypertension, obesity, and metabolic syndrome. Additionally, studies at Yonsei University emphasize the role of inflammation in these pathological processes. In contrast, INSERM’s research on metabolomics and arterial stiffness similarly investigates the influence of inflammation but places stronger emphasis on mechanistic exploration, incorporating key themes related to endothelial function.

The authors ranked among the top 10 in terms of the number of papers published on metabolomics and arterial stiffness are presented in Table 1. Over the past 25 years, Bang-Gee Hsu and Stehouwer Cda have been the most productive author, publishing 19 articles. Scuteri A has the highest number of co-citations, followed by Stehouwer Cda, Ferreira I, and Urbina Em, with all their co-citations exceeding 1,000. The analysis above indicated that their research in this field has garnered widespread attention.

**TABLE 1:** Top 10 authors with the most publications about metabolome and arterial stiffness.

Rank	Author	Country	Article	Total citations	Start year	H index
1	Stehouwer Cda	Netherlands	19	1,403	2002	15
1	Hsu Bg	China	19	319	2013	11
3	Urbina Em	USA	18	1,042	2009	14
4	Zhang Y	China	15	228	2009	10
5	Blacher J	France	14	703	2005	12
5	Ryliskyte L	Lithuania	14	365	2014	8
5	Safar Me	France	14	681	2006	13
5	Shirai K	Japan	14	791	2011	12
5	Tanaka H	USA	14	681	2001	11
10	Benetos A	France	13	603	2006	9
10	Chen Yc	China	13	247	2013	9
10	Ferreira I	Netherlands	13	1,145	2005	13
10	Laucevicius A	Lithuania	13	214	2014	7
10	Scuteri A	Italy	13	1,491	2004	10

USA, United States of America.

### 3.2 Analysis of journal productivity and co-citation

The publications were distributed across 550 journals. Table 2 lists the top 10 journals ranked by publication quantity along with their latest 2023 impact factors (IF). Among these, eight are specialized journals related to cardiovascular health, while two are general journals. The top three prolific journals are the Journal of Hypertension (IF = 3.3), Atherosclerosis (IF = 4.9), and Hypertension (IF = 6.9). There are five publishers from the USA, along with others from the United Kingdom, Switzerland, Italy, and Japan.

**TABLE 2:** Top 10 journals with most articles about metabolome and arterial stiffness.

Rank	Journal	Article	Country	IF	JCR
1	Journal of Hypertension	51	USA	3.3	Q1
2	Atherosclerosis	48	USA	4.9	Q1
3	Hypertension	37	USA	6.9	Q1
4	Journal of Atherosclerosis and Thrombosis	32	Japan	3.0	Q2
5	Hypertension Research	29	Japan	4.3	Q1
6	Cardiovascular Diabetology	26	UK	8.5	Q1
6	American Journal of Hypertension	26	USA	3.2	Q2
8	Nutrition, Metabolism and Cardiovascular Diseases	25	Italy	3.3	Q2
8	Nutrients	25	Switzerland	4.8	Q1
10	Plos One	23	USA	2.9	Q1

UK, United Kingdom.

The top 10 most cited publications, all with more than 330 citations, are listed in Table 3. The article *Predictive validity of health-related fitness in youth: a systematic review*, published in *British Journal of Sports Medicine* in 2009, has received the most citations (701). This study using meta-analysis to find that higher cardiorespiratory fitness in childhood and adolescence is associated with a reduced risk of developing metabolic syndrome and arterial stiffness later in life, underscoring the importance of early-life fitness interventions for long-term cardiovascular health. The paper titled *Insulin resistance, cardiovascular stiffening and cardiovascular disease*, published in the *Metabolism* in 2021, has received the highest average citations per year (84.40). This article summarized that arterial stiffness in obesity and cardiometabolic syndrome (CMS) was driven by hyperinsulinemia and aldosterone-induced activation of serum/glucocorticoid kinase-1 (SGK-1), which disrupted endothelial sodium flux, reduced nitric oxide bioavailability, and promoted cytoskeletal remodeling, positioning SGK-1 as a central mediator linking insulin resistance to vascular stiffening.

**TABLE 3:** The top 10 most cited papers.

Rank	Title	Journal	Year	TC	TC per year	References
1	Predictive validity of health-related fitness in youth: a systematic review	BJSM	2009	701	41.24	(24)
2	Combined Ventricular Systolic and Arterial Stiffening in Patients with Heart Failure and Preserved Ejection Fraction: Implications for Systolic and Diastolic Reserve Limitations	Circulation	2003	695	30.22	(41)
3	Obstructive Sleep Apnea: A Cardiometabolic Risk in Obesity and the Metabolic Syndrome	JACC	2013	549	42.23	(42)
4	Arterial Aging: Is It an Immutable Cardiovascular Risk Factor	Hypertension	2005	513	24.43	(43)
5	Arterial stiffness in diabetes and the metabolic syndrome: a pathway to cardiovascular disease	Diabetologia	2008	428	23.78	(19)
6	Insulin resistance, cardiovascular stiffening and cardiovascular disease	Metabolism	2021	422	84.40	(44)
7	The Effects of Induced Hypogonadism on Arterial Stiffness, Body Composition, and Metabolic Parameters in Males with Prostate Cancer	JCEM	2001	361	14.44	(45)
8	The pathophysiology of hypertension in patients with obesity	Nat. Rev. Endocrinol	2014	354	29.50	(46)
9	Metabolic syndrome amplifies the age-associated increases in vascular thickness and stiffness	JACC	2004	334	15.18	(47)
10	Vascular stiffness mechanoactivates YAP/TAZ-dependent glutaminolysis to drive pulmonary hypertension	JCI	2016	331	33.10	(48)

TC, total citations; BJSM, British Journal of Sports Medicine; JACC, Journal of the American College of Cardiology; JCEM, Journal of Clinical Endocrinology and Metabolism; Nat. Rev. Endocrinol, Nature Reviews Endocrinology; JCI, The Journal of Clinical Investigation.

In addition to high-quality articles focusing on metabolomics in the field of arterial stiffness, literature from other disciplines may help contextualize the research background or aid in interpreting results. We analyzed highly cited references, with Table 4 listing the top 10 most frequently cited works, all with citation frequencies of ≥ 100. The article *Expert consensus document on arterial stiffness: methodological issues and clinical applications*, published in *European Heart Journal* in 2006, was the most cited (284). This study provides a detailed account of the methodologies used to assess arterial stiffness, offering comprehensive background information for another research. It also compiles longitudinal studies on arterial stiffness in European populations, as well as non-pharmacological and pharmacological treatment approaches, thereby serving as an empirical foundation for interpreting findings in other population studies. The most recent research paper among the top 10 cited references is *Arterial Stiffness and Cardiovascular Events: The Framingham Heart Study*, published in *Circulation* in 2010. As part of the classic cardiovascular cohort study, the Framingham Heart Study (FHS), this research followed 2,232 participants over a median follow-up period of 7.8 years. It demonstrated that among various arterial stiffness metrics, pulse wave velocity (PWV)—but not augmentation index, central pulse pressure, or pulse pressure amplification—improved the predictive capacity for cardiovascular disease. Specifically, higher aortic PWV was associated with a 48% increase in cardiovascular disease risk, underscoring the clinical significance of arterial stiffness research and providing a robust foundation for future studies in this field.

**TABLE 4:** The top 10 most cited references.

Rank	Cited reference	Journal	Year	Citations	References
1	Expert consensus document on arterial stiffness: methodological issues and clinical applications	EHJ	2006	284	(25)
2	Prediction of cardiovascular events and all-cause mortality with arterial stiffness: a systematic review and meta-analysis	JACC	2010	201	(49)
3	Aortic stiffness is an independent predictor of all-cause and cardiovascular mortality in hypertensive patients	Hypertension	2001	195	(50)
4	Validity, reproducibility, and clinical significance of non-invasive brachial-ankle pulse wave velocity measurement	Hypertension Research	2002	129	(51)
5	Homeostasis model assessment: insulin resistance and beta-cell function from fasting plasma glucose and insulin concentrations in man	Diabetologia	1985	119	(52)
6	Arterial stiffness and cardiovascular events: the Framingham Heart Study	Circulation	2010	111	(26)
7	Arterial stiffness in diabetes and the metabolic syndrome: a pathway to cardiovascular disease	Diabetologia	2008	109	(19)
8	Aortic pulse-wave velocity and its relationship to mortality in diabetes and glucose intolerance: an integrated index of vascular function	Circulation	2002	104	(53)
9	Mechanisms, pathophysiology, and therapy of arterial stiffness	ATVB	2005	102	(54)
10	Executive Summary of The Third Report of The National Cholesterol Education Program Expert Panel on Detection, Evaluation, And Treatment of High Blood Cholesterol in Adults	JAMA	2001	100	(55)

ATVB, Arteriosclerosis, Thrombosis, and Vascular Biology; EHJ, European Heart Journal; JAMA, Journal of the American Medical Association; NCEP, National Cholesterol Education Program.

### 3.3 Analysis of keywords and abstract

Keywords and the abstract encapsulate the core content of the article and can be utilized to analyze the frontiers in the research field. A total of 2,566 keywords were collected in this study, with 75 of them appearing 10 times or more. Apart from arterial stiffness (*n* = 705) and metabolomics (*n* = 16), the most used term was “pulse wave velocity,” with 301 occurrences. This was followed by “metabolic syndrome” (*n* = 232) and “cardiovascular disease” (*n* = 131).

**FIGURE 3:**
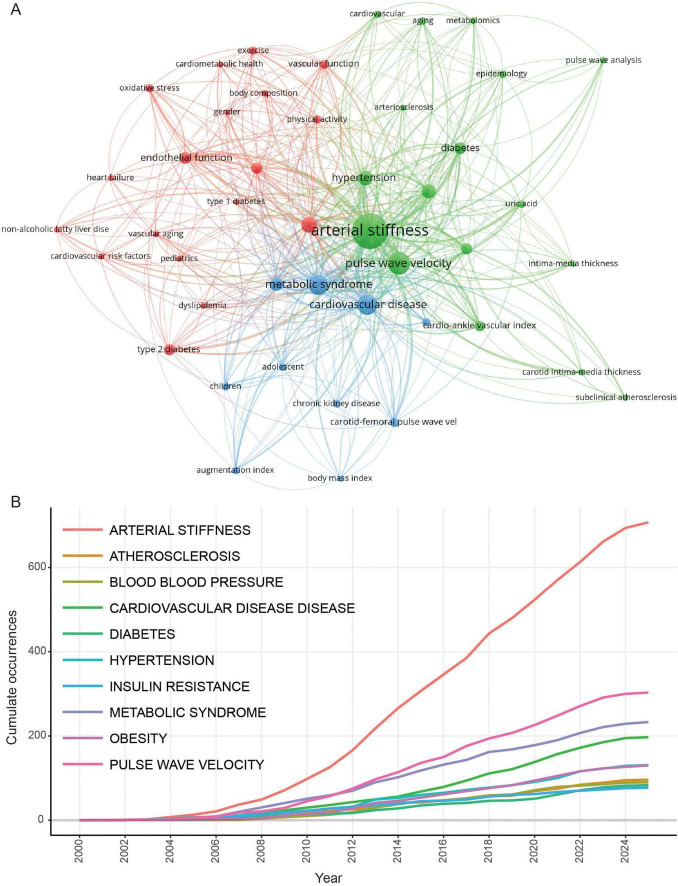
Keywords co-occurrence analysis. **(A)** Keywords co-occurrence network. **(B)** Trending topics of keywords.

As illustrated in the keyword co-occurrence network map (Figure 3A), the keywords were organized into three clusters. The red cluster represents keywords highly associated with arterial stiffness physiological mechanisms, such as “endothelial function,” “oxidative stress,” and others. The green cluster highlight terms linked to “hypertension” and “metabolomics,” including “arteriosclerosis,” “uric acid,” and related concepts. The blue cluster encompasses keywords tied to “children,” “adolescents,” and “body mass index (BMI),” possibly addressing the influence of weight management on arterial stiffness development in in youth and juvenile population. Keyword trend analysis reveals that in 2014 (Figure 3B), the core keyword “arterial stiffness” appeared twice as frequently as the second-ranked keyword (PWV). Subsequent growth rates of “arterial stiffness” surpassed other high-frequency disease-related keywords (e.g., cardiovascular diseases, diabetes, hypertension, and metabolic syndrome), suggesting that research on arterial stiffness as an associated biomarker for metabolic disorders has reached saturation.

**FIGURE 4:**
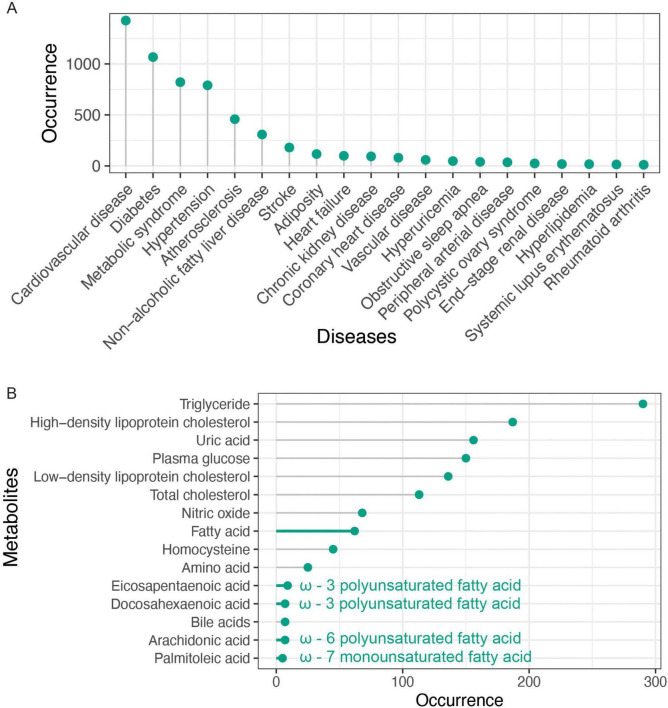
Abstract words frequency analysis. **(A)** Top 20 arterial stiffness-related diseases. **(B)** Top 20 arterial stiffness-related metabolites.

To further understand the research trends, we counted the total number of articles for arterial stiffness-related diseases (Figure 4A). Cardiovascular disease ranks first, with a total of 827 publications. The other diseases among the top 10 are metabolic or specific cardiovascular diseases. Additionally, conditions such as polycystic ovary syndrome, systemic lupus erythematosus, and rheumatoid arthritis are also linked to arterial stiffness. However, research publications in these areas remain limited. Further exploration is warranted to investigate the impact of arterial stiffness on women’s health (including fertility) and whether bidirectional interactions exist between arterial stiffness and autoimmune diseases.

We compiled a list of the 20 most frequently occurring metabolites and, as expected, observed the four lipid parameters and blood glucose (Figure 4B). Moreover, the abstract contained 156 mentions of uric acid, a term that frequently appeared in the keywords. Notably, we identified an association between arterial stiffness and fatty acids (Table 5), specifically highlighting four key unsaturated fatty acids: arachidonic acid, docosahexaenoic acid (DHA), eicosapentaenoic acid (EPA), and palmitoleic acid, whose shared unsaturated structure suggests broader implications for other unsaturated fatty acids in arterial stiffness. Simultaneously, bile acids—a metabolically diverse category with functional overlaps to fatty acids—emerge as a promising yet underexplored area, evidenced by their strikingly lower research term frequency compared to fatty acids (7 vs. 62), underscoring the need for deeper mechanistic investigations into their role in vascular health. Considering that gut microbiota is involved in the metabolism of fatty acids and bile acids, we analyzed the occurrence of gut microbiota in the abstract. Though it was only mentioned 34 times, suggesting that investigating metabolites associated with arterial stiffness from the standpoint of gut microbiota metabolites has attracted a lot of interest. This connection emphasizes the potential of gut microbiota-derived metabolites as biomarkers in understanding and addressing arterial stiffness.

**TABLE 5:** Hallmarks of arterial stiffness-related metabolites.

Metabolites class	Metabolites	Species	Vascular effects	References
Fatty acid	Arachidonic acid	Rats	Augmented AA-induced vasoconstriction and COX 1-mediated arterial stiffness in high-fat diet	(56)
		Humans	Improved arterial stiffness via elevated EPA/AA ratio and attenuated inflammation; exacerbated arterial stiffening with increased AA/LA ratio	(57, 58)
	Docosahexaenoic acid	Mice	Reduced wall shear stress, oscillatory shear, and IL-1β-mediated inflammation; attenuated atherosclerosis progression and aortic stiffness via indirect IL-1 inhibition	(59)
		Rats	Reduced arterial stiffness, oxidative stress, and endothelial lipotoxicity; enhanced NO-induced vasodilation, VSMC potassium channel activation, and autophagy-mediated vascular protection	(60, 61)
		Humans	Unchanged endothelial function and arterial stiffness in healthy youth; improved pulse wave velocity, inflammation, and lipid profiles in metabolic syndrome with low-dose, short-term omega-3 supplementation	(62–64)
	Eicosapentaenoic acid	Mice	Reduced arterial stiffness, post-MI cardiac remodeling, and M1 macrophage polarization; enhanced TRPV4-mediated vasodilation and desensitization resistance via non-KATP channel pathways	(65–67)
		Rats	Reduced oxidative stress, endothelial dysfunction, and medial calcification; enhanced NO-induced vasodilation, macrophage polarization, and MMP-9 inhibition in hypertensive and calcified arteries	(60, 68, 69)
		Humans	Reduced arterial stiffness, vascular reflected wave amplitude, and residual cardiovascular risk; enhanced EPA/AA ratio, adiponectin-mediated anti-inflammation, and plaque stabilization via triglyceride-rich lipoprotein modulation	(57, 70, 71)
	Palmitoleic acid	Mice	Reduced carotid artery stiffening, plaque progression, and MMP-9 hyperactivity; enhanced cerebrovascular compliance, eNOS-dependent dilation, and endothelial resilience	(72)
		Rats	Prevented menopause-associated arterial stiffness, vascular insulin resistance, and endothelial dysfunction through PI3K/Akt pathway activation and reduced oxidative-inflammatory stress	(73–75)
Bile acid	Primary bile acid	Humans	Attenuated FGF19-regulated bile acid homeostasis with exacerbated arterial stiffness, atherogenic dyslipidemia, and vascular metabolic dysfunction in type 2 diabetes	(76)
	Secondary bile acid: deoxycholic acid	Humans	Elevated chronic kidney disease-associated coronary artery calcification and vascular stiffening	(77)
	Secondary bile acid: tauroursodeoxycholic acid	Mice	Attenuated diabetes-associated arterial stiffness, endothelial dysfunction, and endoplasmic reticulum stress	(78)

AA, arachidonic acid; COX 1, cyclooxygenase enzymes 1; EPA, eicosapentaenoic acid; LA, linoleic acid; IL, interleukin; NO, nitric oxide; VSMC, vascular smooth muscle cell, MI, myocardial infarction; KATP, potassium-adenosine triphosphate; MMP, matrix-metalloproteinase; eNOS, endothelial nitric oxide synthase; PI3K, phosphatidylinositol 3-kinase; FGF19, fibroblast growth factor 19.

## 4 Discussion

This bibliometric investigation analyzed 1,654 Web of Science Core Collection records (2000–2025.03) to map the evolving landscape of metabolomics research in arterial stiffness. Utilizing Bibliometrix and VOSviewer, we systematically quantified temporal publication trends, geographic productivity distributions, and institutional collaboration networks. The methodology synergized quantitative bibliometric indicators with text-mining techniques, identifying fatty acids and bile acids metabolism as predominant research foci. Our tool selection adhered to established protocols for metabolomics research evaluation, building upon Yu group’s validated bibliometric analysis framework for cardiovascular disease (18).

In this research field, studies growth can be categorized into three stages: a slow growth phase before 2006, a rapid growth phase beginning in 2006, and a steady growth phase starting in 2013. From the perspective of spatial distribution, the top 10 countries accounted for 67.4% of the total publications. The USA leads in this area, producing the highest number of publications and having the most international collaborations. The USA holds a leading role and represents the global frontier in this field. In addition to its advanced omics testing equipment, significant investment in healthcare has also contributed to its success. China, with strong economic conditions, ranks second in publication volume. Austria ranks 25th in the number of publications, but it has the highest average citation count, exceeding 60. The average number of citations in the USA is still in the top 10, whereas China is ranked below 10th, suggesting that the average quality of articles from China still needs to be improved. Therefore, this field continues to receive economic support from various countries, and international collaboration has facilitated the application of metabolomics in the study of arterial stiffness.

The top 10 institutions by publication volume are from six different countries, indicating that research in this field is quite widespread. Given that the United States is the country with the highest number of publications, it is not surprising that two of the top 10 institutions are from the USA. Interestingly, three of France’s institutions rank among the top 10 despite the country being tenth in terms of publication output. This might be attributed to a more focused research effort among connected teams. Surprisingly, China and Japan, which rank second and third in publication volume, have no institutions in the top ten. The authors with the highest publication output show some variation in institutional affiliation; in addition to the USA and France, two Chinese scientists and two Lithuanian scientists are also actively researching in this field. The earliest among them began related research in 2001, while the latest entered the field around 2014, which marks a turning point in the publication timeline.

The effect of arterial stiffness has been significantly advanced by seminal work from highly cited researchers. Stehouwer Cda is an expert in metabolic diseases, who summarized that arterial stiffness is a significant indicator of type 2 diabetes and metabolic syndrome, suggesting a close association between arterial stiffness and metabolism, later confirming role of arterial stiffness in microangiopathic encephalopathy via multi-studies (19, 20). Hsu BG epidemiologically established arterial stiffness associations with renal biomarkers (creatinine/cystatin C) through longitudinal cohorts (21). Complementary insights from Urbina EM revealed youth-specific glucose-lipid profiles and type 1 diabetes correlations (22, 23), collectively reshaping clinical understanding for vascular-metabolic disease.

High citations and co-citations studies highlights key insights from highly cited arterial stiffness research: the 2009 systematic review (24) emphasizes early-life fitness interventions for long-term cardiovascular health, while the 2021 *Metabolism* article identifies SGK-1 as a critical mediator linking insulin resistance to vascular stiffening (19). Cross-disciplinary references, such as the 2006 methodological consensus *(25)* and 2010 Framingham Study (26), underscore the importance of standardized assessments and hemodynamic metrics like PWV. These findings reveal dual research trajectories—mechanistic exploration and clinical validation—that future studies should integrate to bridge molecular pathways with diagnostic tools.

Keywords and abstracts in the literature help identify research hotspots in this field. The main keywords of the current studies include “uric acid,” “C-reactive protein,” “endothelial function,” “insulin resistance,” “inflammation,” “oxidative stress,” “metabolic syndrome,” and “non-alcoholic fatty liver disease,” among others. From the perspective of research subjects, the adolescents is highly regarded. In recent years, adolescents, particularly those with obesity, have garnered attention from researchers, which aligns with global trends. According to a report published by the NCD Risk Factor Collaboration (27), there are currently 159 million adolescents aged 5–19 who are affected by obesity, with the prevalence increasing from 4% in 1975 to 20% in 2022. In terms of diseases under investigation, the most common ones are metabolic syndrome and cardiovascular disease. Currently, diseases like hyperuricemia and non-alcoholic fatty liver disease are rarely studied in relation to arterial stiffness and other comorbidities of metabolic disorders. In a follow-up study involving 3,763 East Asians aged 30–70, it was discovered that hyperuricemia and higher serum uric acid were independent risk factors for arterial stiffness (28). Additionally, hyperuricemia comorbidities with elevated arterial stiffness will exacerbate the decline in kidney function (29). While no studies have directly investigated the association between hyperuricemia and arterial stiffness in adolescents, we believe this relationship warrants further attention. Two epidemiological studies from hospitals in East Asia reported a surprisingly high prevalence of hyperuricemia among adolescents (30, 31). One hospital report on 9,371 teenagers between the ages of 13 and 19 revealed that 25.4% of them had hyperuricemia (serum uric acid levels ≥ 7 mg/dL). For non-alcoholic fatty liver disease, it is not only positively correlated with arterial stiffness (32), but it also has a causal association with arterial stiffness in a Mendelian randomization study involving 127,121 participants, where arterial stiffness was the outcome variable (33). A limited number of studies mentioned autoimmune diseases, polycystic ovarian syndrome, and obstructive sleep apnea. These associations are not all positive results and require more in-depth research. For example, in the case of polycystic ovary syndrome, some studies report that women with this condition have relatively higher arterial stiffness (34, 35), while another study have found no differences (36).

From a metabolomics perspective, in addition to lipids, fatty acids and bile acids deserve in-depth study as potential biomarkers. Clinical trials have found that Omega-3 polyunsaturated fatty acids such as EPA can improve arterial stiffness (37). One of secondary bile acid, lithocholic acid, has been shown to be a calorie restriction metabolite, extends lifespan and enhances healthspan by activating AMPK (38). On the other hand, combined with metagenomic studies, it was found that specific short-chain fatty acid-producing bacteria species ameliorated atherosclerosis in mice (39). Research on the gut microbiota has yielded impactful findings, reflected in the high average citation rates of related publications. Microbes are intimately involved in a wide range of metabolic pathways. For example, by inhibiting TMAO synthesis in mice, researchers have shown that it is possible to reduce arterial stiffness and improve cardiovascular health (40).

Though this bibliometric analysis represents the first comprehensive exploration of research trends and hotspots within the field of metabolomics in arterial stiffness, it is important to acknowledge certain limitations. The inclusion criteria were limited to English-language publications, which may have inadvertently overlooked significant contributions from non-English-speaking researchers.

The bibliometric analysis presented herein highlights the substantial research value and broad applicability of metabolomics in the study of arterial stiffness. Future metabolomics research on arterial stiffness could achieve innovation through strategic cohort selection and metabolite prioritization. For population-based studies, metabolically healthy cohorts such as disease-free adolescents or adults with autoimmune conditions (e.g., systemic lupus erythematosus) may reveal early biomarkers preceding metabolic dysregulation. Mechanistically, conservative approaches should focus on validating unsaturated fatty acids (e.g., DHA, EPA) while prioritizing secondary bile acid metabolites, given their emerging mechanistic links to vascular function. More exploratory strategies could target understudied gut microbial metabolites, particularly tryptophan derivatives (e.g., indole derivatives). By focusing on these critical areas, researchers may further advance our understanding of arterial stiffness and develop more effective interventions to mitigate its associated cardiovascular risks.

## Data Availability

Publicly available datasets were analyzed in this study. This data can be found here: Publicly available datasets were analyzed in this study. This data can be found at: Web of Science.
